# Antibacterial Biomaterials for Medical Applications: From Materials Design to Functional Synergy

**DOI:** 10.3390/jfb17100519

**Published:** 2026-10-15

**Authors:** Yueming Wu, Min Zhou

**Affiliations:** 1 Global Institute of Future Technology (GIFT), Shanghai Jiao Tong University, Shanghai 200240, China; 2 State Key Laboratory of Bioreactor Engineering, School of Materials Science and Engineering, East China University of Science and Technology, Shanghai 200237, China

Biomaterials are widely used in medical devices, tissue repair, orthopedic and dental implants, wound management, and localized drug delivery. However, microbial adhesion, colonization, and biofilm formation remain major challenges that can compromise the safety and clinical performance of biomaterials. These concerns are particularly pressing in the context of increasingly complex infections and antimicrobial resistance. Therefore, developing biomaterials with effective and safe antimicrobial functions, while preserving biocompatibility, tissue integration, and therapeutic performance, has become an important research priority. This Special Issue comprises nine original research articles and one review, covering silver-based systems, bioactive inorganic materials, implant surface engineering, and nanocarrier-based delivery platforms. It highlights emerging strategies that bridge materials design and functional synergy.

The composition and intrinsic properties of biomaterials provide the foundation for antimicrobial activity. Du et al. reviewed the development and biomedical applications of silver and silver-based materials in wound management, while Holguín-Meráz et al. demonstrated the influence of silver nanoparticle size on oral biofilm inhibition. Wang et al. further integrated ultralow-dose silver nanoparticles with TiO_2_ nanotubes to combine infection control with immunomodulation and bone regeneration [1,2,3]. Rajkumar et al. elucidated the bactericidal mechanisms of S53P4 bioactive glass, highlighting the roles of ion release, membrane damage, and metabolic disruption, whereas Huang et al. improved the handling properties and antibacterial performance of premixed calcium silicate cement through compositional optimization [4,5]. Collectively, these studies demonstrate that antimicrobial performance is governed not only by the active component itself but also by material composition, dose, carrier architecture, and dissolution or release kinetics, underscoring a shift from simply introducing antimicrobial activities toward functional synergy.

At the material interface, surface chemistry and physical structure provide complementary means of regulating microbial interactions and host responses. Dorner-Reisel et al. showed that fullerene C_60_ films and tribomechanical surface patterning conferred moderate antibacterial activity against Staphylococcus aureus on zirconia, whereas no comparable effect was observed against Escherichia coli, highlighting the species-dependent nature of surface-mediated antimicrobial activity [6]. Liu et al. developed mussel-inspired catechol-terminated quaternary ammonium compounds for facile titanium surface modification and demonstrated that antimicrobial activity could be tuned by alkyl chain length and coating strategy [7]. Iusan et al. further showed that nanostructured titanium surfaces could simultaneously reduce bacterial proliferation and biofilm formation while promoting osteoblastic adhesion and viability, illustrating the potential of physical surface design to couple infection control with osseointegration [8]. Beyond surface engineering, nanocarrier systems enable localized control over the stability, presentation, and release of antimicrobial agents. Tsamesidis et al. used Ca/Ce-doped mesoporous silica nanoparticles to enhance the loading and sustained release of Artemisia absinthium extract, whereas Sundar et al. developed a liposomal gel co-delivering silver nanoparticles and ibrexafungerp citrate for topical treatment of vulvovaginal candidiasis [9,10]. Together, these studies highlight complementary approaches spanning surface chemistry, physical topography, and localized or combination delivery.

Future advances will require a shift from simply achieving antimicrobial efficacy toward developing safe, durable, and clinically relevant functional synergy. This will require standardized and physiologically relevant evaluation models that better capture biofilms, polymicrobial communities, and dynamic biological environments, together with integrated assessment of antimicrobial activity, biocompatibility, immune responses, tissue function, long-term stability, and potential resistance. Reproducible fabrication, scalable manufacturing, and rigorous quality control will also be essential for clinical translation. It is anticipated that future biomaterials will evolve from simple antimicrobial materials into systems that integrate multiple functions, such as antimicrobial activity, tissue regulation, and local drug delivery. However, how the integration of these functions affects antimicrobial activity and overall therapeutic efficacy in vivo remains to be further investigated.

## Data Availability

No new data were created or analyzed in this study. Data sharing is not applicable to this article.
